# METTL3-mediated m ^6^A modification of pri-miRNA-31 promotes hypertrophic scar progression: METTL3 drives hypertrophic scar progression via pri-miRNA-31

**DOI:** 10.3724/abbs.2025033

**Published:** 2025-03-18

**Authors:** Qirui Wang, Jialin Hou, Siyi Zeng, Xue Wang, Yimin Liang, Renpeng Zhou

**Affiliations:** 1 Department of Plastic and Reconstructive Surgery Shanghai Ninth People’s Hospital Shanghai Jiao Tong University School of Medicine Shanghai 200011 China; 2 Department of Dermatology Shanghai Ninth People’s Hospital Shanghai Jiao Tong University School of Medicine Shanghai 200011 China

**Keywords:** hypertrophic scar, METTL3, m ^6^A modification, microRNA31, ZBTB20

## Abstract

Hypertrophic scar (HS) is a pathological scar characterized by excessive dermal fibrosis. Aberrant m ^6^A modification patterns have been identified in HS; however, the expression of the methyltransferase, along with its function and molecular mechanisms in HS, remains unclear. In this study, we find that both the protein level of METTL3 and the level of m6A methylation are upregulated in HS compared with normal skin. To investigate the role of METTL3 in HS, we knock down *METTL3* in HS-derived fibroblasts (HSFBs) via shRNA. *METTL3* knockdown reduces the expressions of collagen types I and III (COL I/III) and α-SMA, inhibits cell proliferation and migration, and induces cell cycle arrest in the G1 phase. MeRIP-seq analysis reveals m ^6^A modification sites on pri-miR-31. Our data indicate that the expression level of pri-miR-31 is elevated in *METTL3*-knockdown HSFBs, whereas the level of mature miR-31-5p is reduced. Notably, transfection of a miR-31-5p mimic into HSFBs partially counteracts the inhibitory effects of the m ^6^A methylation inhibitors cycloleucine and STM2457 (a specific inhibitor of METTL3) on fibrosis and cellular proliferation. Additionally, we confirm that ZBTB20 is a downstream target of miR-31-5p and that knockdown of *ZBTB20* inhibits fibroblast fibrosis. Collectively, our findings elucidate the epigenetic mechanism of METTL3/m ^6^A/pri-miR-31/ZBTB20 in HS fibrosis, providing a potential therapeutic target for HS.

## Introduction

Hypertrophic scar (HS) is a pathological scar characterized by abnormal dermal fibrosis [1]. HS can lead to symptoms such as pruritus and joint contracture [2]. The pathogenesis of HS involves excessive proliferation of fibroblasts, increased synthesis and deposition of collagen [3]. However, the molecular mechanisms underlying fibrosis in HS remain inadequately understood. 

N6-methyladenosine (m ^6^A) is the most abundant RNA modification in mammals [4]. This methylation is mediated by the methyltransferase complex, which is primarily composed of methyltransferase-like 14 (METTL14), METTL3 and Wilms tumor 1-associated protein (WTAP), and other components [5]. Previous studies have shown that m ^6^A modification plays a crucial role in the development and progression of various diseases [ 6, 7]. M ^6^A modifications are found not only in mRNAs but also in non-coding RNAs, such as microRNAs (miRNAs) [8]. Liu *et al*. [9] found that alterations in m ^6^A modification patterns during HS formation are associated with fibrosis-related pathways. However, the specific mechanisms by which m ^6^A modifications regulate HS formation remain unclear. 

MicroRNAs (miRNAs) are short, single-stranded non-coding RNA molecules consisting of 19 to 25 nucleotides [10]. Generally, miRNAs regulate the post-transcriptional silencing of target genes [10]. The maturation of miRNAs involves the processing of primary miRNAs (pri-miRNAs) by microprocessor complexes [11]. METTL3 participates in pri-miRNA processing and miRNA maturation through m ^6^A modifications [12]. We previously demonstrated higher expression of hsa-miRNA-31-5p in HS tissues than in normal skin [13]. Here, both m ^6^A modification levels and METTL3 expression were elevated in HS tissues compared with normal skin. *METTL3* knockdown in HS fibroblasts reduced collagen synthesis and significantly affected cell proliferation and migration. Furthermore, METTL3 promoted the maturation of pri-miR-31 in HS fibroblasts in an m ^6^A-dependent manner. Moreover, miR-31-5p modulated the functions of HS fibroblasts by silencing *ZBTB20*. Overall, this study reveals the potential role of METTL3 in the pathogenesis of HS and provides evidence that METTL3 promotes the maturation of pri-miR-31 via m ^6^A modification. 

## Materials and Methods

### Patients and samples

This study was approved by the Ethics Committee of the Ninth People’s Hospital (SH9H-2020-TK234-1) (Shanghai, China), and informed consent was obtained from each patient. A total of 8 hypertrophic scar (HS) tissues and 8 adjacent normal skin (NS) tissues were collected from the Department of Plastic Surgery at the Ninth People’s Hospital. The samples were obtained from the neck, face, upper extremities and popliteal fossa of five males and three females. Parts of the samples were used to isolate primary skin fibroblasts, while other parts were fixed in 4% paraformaldehyde for immunohistochemical analysis. Additional samples were rapidly frozen in liquid nitrogen for protein and RNA extraction.

### Isolation and culture of HS/NS dermal fibroblasts

First, the NS or HS tissues were washed three times with phosphate-buffered saline (PBS) containing a penicillin-streptomycin solution (C0222; Beyotime, Shanghai, China). The subcutaneous tissue was excised, and the dermal tissue was minced as finely as possible. The minced tissue was digested with 0.3% type I collagenase (SCR103; Sigma‒Aldrich, St Louis, USA) at 37°C for 5 h at 200 rpm. After digestion, the tissue was filtered through a sterile 70-μm filter, followed by centrifugation at 500 *g* for 5 min, and the supernatant was discarded. The remaining pellet was re-suspended in Dulbecco’s modified Eagle’s medium (DMEM; Gibco, Carlsbad, USA) supplemented with 10% fetal bovine serum (FBS; Gibco). The cells were cultured at 37°C in a 5% CO _2_ incubator. Fibroblasts from the 2 ^nd^ to 5 ^th^ passages were used for subsequent experiments. 

### Cell treatment

To inhibit m ^6^A methylation, the cells were exposed to 40 μM cycloleucine (CL, HY-30008; MCE, Monmouth Junction, USA) for 48 h. Additionally, to inhibit METTL3, the cells were treated with 20 μM STM2457 (S9870; Selleck, Huston, USA) or a control (DMSO equivalent) for 24 h. 

### Cell transfection

Short hairpin RNAs (shRNAs) were subsequently cloned and inserted into the lentiviral vector pLKO.1 (Plasmid 10878; Addgene, Watertown, USA). The sequences of shRNAs used in this study were as follows: scrambled shRNA: 5′-ACTCAAAAGGAAGTGACA AGA-3′; METTL3 shRNA1: 5′-GCTGCACTTCAGACGAATT-3′; and METTL3 shRNA2: 5′-GCCAAGGAACAATCCATTGT-3′. To generate lentiviral particles, HEK293 cells were transfected with the pCMV-VSVG vector (Addgene), the pLKO.1 shRNA vector, or the pCMV-dR8.9 vector (Addgene). HS-derived fibroblasts (HSFBs) were then transfected with lentiviral supernatant containing 8 μg/mL polybrene (40804ES76; Yeasen, Shanghai, China) for 24 h. The cells were subsequently selected with 1 μg/mL puromycin (A1113803; Gibco).

Additionally, we transfected miR-31-5p mimics or negative controls (NCs) into NS-derived fibroblasts (NSFBs) via LipoRNAi transfection reagents (C0535; Beyotime). The sequences of the has-miR-31-5p mimics/NC (synthesized by Genomeditech, Shanghai, China) are as follows: has-miR-31-5p mimics: 5′-AGGCAAGAUGCUGGCAUAGCU-3′, 5′-AGCUAUGCCAGCAUCUUGCCU-3′; and has-miR-31-5p mimics NCs: 5′-UCACAACCUCCUAGAAAGAGUAGA-3′, 5′-UCUACUCUUUCUAGGAGGUUGUGA-3′.

Furthermore, we transfected ZBTB20 siRNAs and their NCs into human skin fibroblasts (HSFBs) via LipoRNAi transfection reagents. The sequences synthesized by Genomeditech are as follows: si-ZBTB20-1#: sense 5′-GGAUCUACUCGGCACUCUATT-3′; antisense 5′-UAGAGUGCCGAGUAGAUCCTT-3′; si-ZBTB20-2#: sense 5′-GGAUCACACGCAUCCAUGATT-3′; antisense 5′-UCAUGGAUGCGUGUGAUCCTT-3′; si-ZBTB20-NC: sense 5′-UUCUCCGAACGUGUCACGUTT-3′; antisense 5′-ACGUGACACGUUCGGAGAATT-3′.

### m ^6^A dot-blot analysis 

m ^6^A dot-blot analysis was performed to detect the m ^6^A levels in total RNA. The methods followed the protocol we previously described [14]. Briefly, total RNA was extracted via TRIzol (15596--026; Thermo Fisher Scientific, Waltham, USA). The mRNA was extracted via the Dynabeads mRNA purification Kit (Sigma-Aldrich). Then, 40 ng of mRNA was used for dot blot analysis. After UV crosslinking and blocking with 5% milk/TBST, the membrane was incubated with an antibody specific for m ^6^A (ab284130; Abcam, Cambridge, UK). The signal intensity was quantified via ImageJ software. Methylene blue staining was used to confirm equal loading of mRNA. 

### Western blot analysis

RIPA lysis buffer (Beyotime) was used to extract proteins from fibroblasts in the HS and NS groups. SDS–polyacrylamide gel electrophoresis (SDS-PAGE) protein loading buffer (TransGen, Beijing, China) was added to the samples, which were subsequently boiled. Proteins were separated via 4%‒20% SDS-PAGE and transferred to NC membranes. The membranes were blocked with 5% milk/TBST and incubated overnight at 4°C with the corresponding primary antibodies. After washing three times with TBST containing 0.1% Tween-20, the corresponding secondary antibodies were used to incubate with the membranes for 1 h at room temperature, followed by additional washing. Finally, ECL luminous mixture (P0018M; Beyotime) was used to detect the proteins. The primary antibodies used were as follows: METTL3 (ab195352, 1:500; Abcam), β-actin (sc-58673, 1:500; Santa Cruz, Santa Cruz, USA), COL I (ab138492, 1:500; Abcam), COL III (ab184993, 1:500; Abcam), α-SMA (ab7817, 1:750; Abcam), ZBTB20 (ab127702, 1:300; ABclonal, Wuhan, China), SEPHS1 (A13403, 1:300; ABclonal), LITAF (A5469, 1:300; ABclonal), and GAPDH (60004-1-Ig, 1:10000; Proteintech, Rosemont, USA). The data were analyzed using ImageJ software (NIH, Bethesda, USA).

### RNA extraction and quantitative real-time PCR (qRT-PCR)

Total RNA was extracted using TRIzol reagent (Invitrogen, Carlsbad, USA). To measure the primary miRNA transcripts, the TaqMan Pri-miRNA assay (4427012, pri-miR-31: Hs03302684_pri; Thermo Fisher Scientific) was utilized according to the manufacturer’s instructions. To quantify the expression of miR-31-5p, the miRcute Plus miRNA First-Strand cDNA Kit (TIANGEN, Beijing, China) and the miRcute Plus miRNA qPCR Kit (SYBR Green; TIANGEN) were used. Other mRNAs were reverse transcribed into cDNA via ABScript III RT Master Mix (ABclonal). The quantification of mRNA was performed using Universal SYBR Green Fast qPCR Mix (ABclonal). The primers used are listed in Table 1.

**Table:** **Table 1** The sequences of primers used in this study

Gene	Primer sequence (5′→3′)
*β-Actin*	Forward Reverse	GGACTTCGAGCAAGAGATGG AGCACTGTGTTGGCGTACAG
*U6*	Forward Reverse	CTCGCTTCGGCAGCACA AACGCTTCACGAATTTGCGT
*COL1A1*	Forward Reverse	GAGGGCAACAGCAGGTTCACTTA TCAGCACCACCGATGTCCA
*COL3A1*	Forward Reverse	CCACGGAAACACTGGTGGAC GCCAGCTGCACATCAAGGAC
*α-SMA*	Forward Reverse	GACAATGGCTCTGGGCTCTGTAA TGTGCTTCGTCACCCACGTA
*hsa-miR-31*	Forward Reverse	GCAAGATGCTGGCATAG GAACATGTCTGCGTATCTC
*ZBTB20*	Forward Reverse	CTCTGCAACAAGACTTTCACCGC AGGAGAAGGAGCGCCAACAGAT
*LITAF*	Forward Reverse	CCTGATGGGAAGGGCATGAATC GATAGGGCGGTCCAAAAAGGTG
*SEPHS1*	Forward Reverse	GTGTCATTCCTTTGAGGCACGG AGGTCACTGAGGACATTGGCAC

### Immunohistochemical (IHC) staining

Six samples were analyzed using immunohistochemical staining. Fixed tissues were embedded in paraffin and sectioned into 5-μm slices. The sections were subjected to heat-induced antigen retrieval and permeabilized with 0.1% Triton X-100. After being blocked with 10% donkey serum, the sections were incubated with the primary antibody overnight at 4°C. The following day, the sections were rinsed with PBS three times, incubated with a secondary antibody at room temperature for 1 h, and washed again with PBS three times. DAB chromatographic detection was performed, followed by hematoxylin staining. The antibodies used included rabbit monoclonal antibody against METTL3 (ab195352, 1:100; Abcam) and goat anti-rabbit IgG-HRP (A0208, 1:50; Beyotime). To quantify the percentage of positive cells, at least five randomly selected fields were analyzed for the number of cells. The DAB-stained cells and total cells were counted via Photoshop CS6 (Adobe Systems, San Jose, USA) with a counting tool.

### Dual-luciferase reporter assay

The ZBTB20-wild-type (WT)/ZBTB20-mutant (MUT, binding site mutant in the 3′UTR sequence, purchased from Genomeditech) vector and hsa-miR-31-5p mimics/NC were co-transfected into HEK293 cells via Lipofectamine 2000 (11668500; Invitrogen). The vector used was GM-1013FL02:PGL3-CMV-LUC-MCS (Genomeditech). After 48 h, the cells were lysed with cell lysis buffer. A dual-luciferase reporter assay system (Genomeditech) was used to measure luciferase activity.

### Transwell migration assay

Transfected HSFBs were assessed for migration using 24-well Transwell plates (Corning, Steuben County, USA) with 8-μm pore size inserts. The transfected HSFBs were seeded at a density of 1000 cells per well and cultured with serum-free DMEM in the upper chamber, while the lower chambers contained DMEM containing 10% FBS. The cells were cultured in a CO _2_ incubator at 37°C for 48 h. The upper chamber was subsequently removed, and the non-migrated cells on the upper side were discarded. The migrated cells on the lower side were fixed with 4% paraformaldehyde and stained with 0.1% crystal violet. Nine fields were randomly selected, and the average number of cells was determined via ImageJ software. 

### Cell proliferation analysis

Cell proliferation analysis in this study included MTT, CCK-8 and EdU assays. For the MTT assay, HSFBs were seeded in 96-well plates at 1000 cells per well. Then, 20 μL of 5 mg/mL MTT solution (Sigma-Aldrich) was added to each well, and the mixture was incubated for 4 h. After the reaction mixture was removed, 100 μL of DMSO was added to each well. The absorbance was measured at 570 nm at 24, 48 and 72 h.

For the CCK-8 assay, cells transfected with the miR-31-5p mimic or NC were seeded in 96-well plates and cultured for 24, 48, 72 or 96 h. After incubation, 10 μL of CCK-8 reagent (Sigma-Aldrich) was added to each well. The cells were incubated at 37°C for 2 h before the absorbance was measured at 450 nm.

For EdU staining, transfected cells were seeded in 24-well plates and cultured in DMEM containing 10% FBS. The cells were then incubated with EdU reagent (Beyotime) for 2 h, fixed with 4% paraformaldehyde for 10 min, and then permeabilized with 0.3% Triton X-100 for 10 min. Following the instructions of the EdU staining kit (Beyotime), the nuclei were stained with Hoechst.

### Cell cycle analysis

Transfected HSFBs were fixed with 70% ethanol at 4°C for 30 min and then rinsed with PBS. The cells were treated according to the instructions of the cell cycle and apoptosis detection kit (Beyotime) with propidium iodide (PI) containing RNase A and incubated at 37°C for 30 min. Finally, flow cytometry was used for analysis.

### RNA-seq, m ^6^A-modified RNA immunoprecipitation-seq (MeRIP-seq) analysis 

RNA-seq was performed by GENEWIZ, Inc. (Suzhou, China), involving RNA extraction, quality detection, library construction and high-throughput sequencing. RNA-seq was performed on *METTL3*-knockdown and control HSFBs using the Illumina platform. In this study, DESeq2 was used for differential gene analysis. A log2|fold change| > 3 and a *P*_ adj_ value < 0.05 were considered statistically significant. 

MeRIP followed the m ^6^A-seq protocol [15], involving RNA purification, fragmentation into 100 nt fragments and capture with anti-m ^6^A antibodies. After elution, a library was constructed and sequenced. Peak and motif analyses were performed via MACS2 and HOMER software. 

### Statistical analysis

Statistical analyses were performed and charts were generated with GraphPad Prism 8.0. The experiments were repeated three times, and the data are expressed as the means ± standard deviations (SDs). Student’s *t* test was used to compare two groups. Differences among multiple groups were compared via one-way ANOVA or two-way ANOVA, followed by the Bonferroni post hoc correction. A *P* value less than 0.05 was considered statistically significant. 

## Results

### Elevated m ^6^A methylation and METTL3 levels in hypertrophic scar tissue 

**Figure 1:**
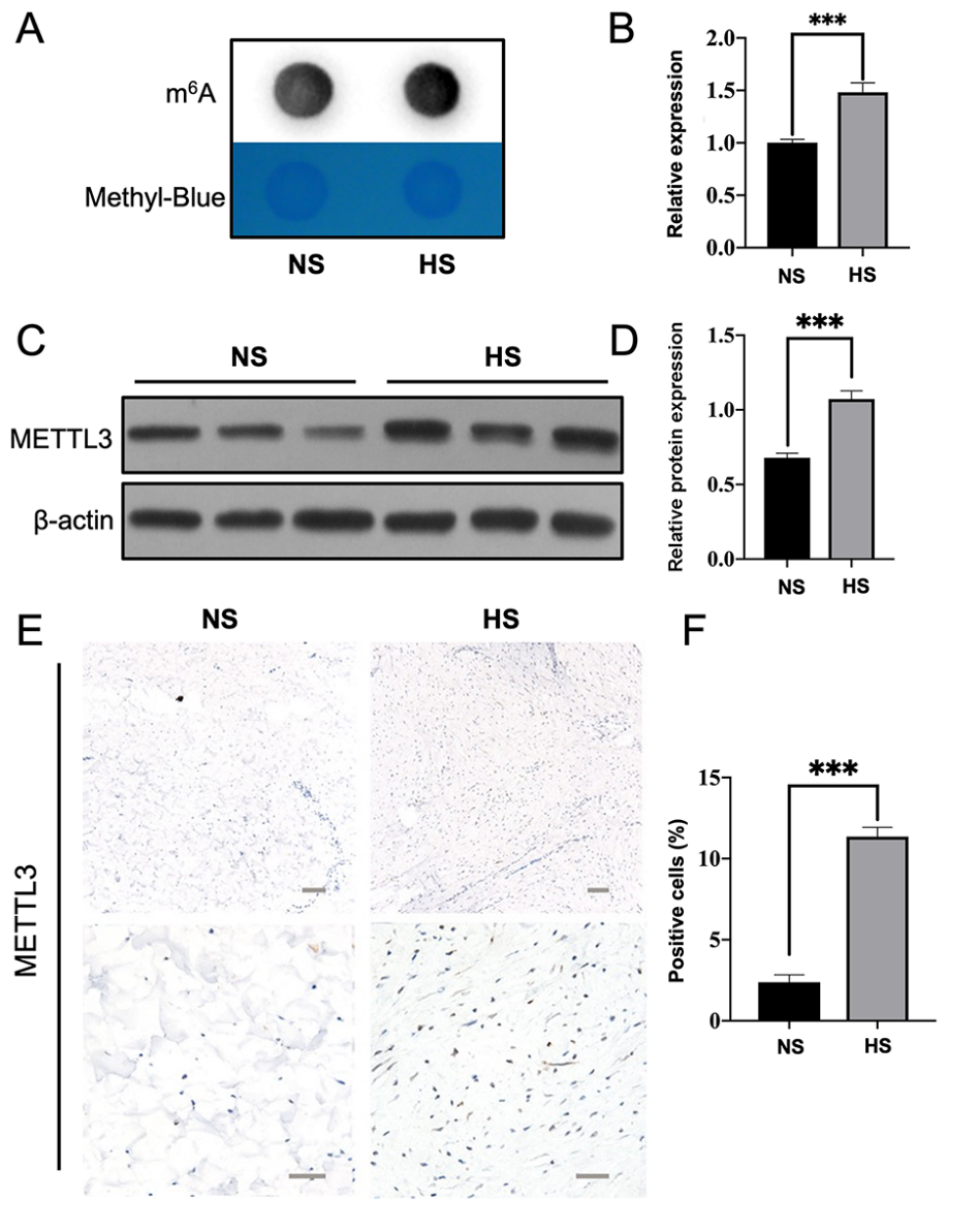
Figure 1 Elevated m ^6^A methylation levels and METTL3 expression in HS (A) m6A dot-blot analysis showing m6A modification levels in HS. (B) Grayscale analysis of the m6A dot-blot results. (C) The protein expression level of METTL3 was elevated in the HS group compared with the NS group, as detected by western blot analysis. (D) Grayscale analysis of METTL3 protein expression. (E) Immunohistochemical staining for METTL3 in HS and NS samples (upper panel, low-magnification field; lower panel, high-magnification field). (F) Quantification of METTL3-positive cells in NS and HS tissues at low magnification. NS, normal skin; HS, hypertrophic scar. Scale bar: 50 μm. ***P < 0.001.

The m ^6^A dot blot analysis showed significantly higher m ^6^A levels in HS tissue compared to normal skin ( Figure 1A,B). The western blot analysis results showed elevated levels of METTL3 in HS tissues ( Figure 1C,D). Furthermore, IHC staining demonstrated a higher proportion of METTL3-positive cells in HS tissue than in normal skin ( Figure 1E,F).

### Silencing of *METTL3* inhibits the fibrosis, proliferation and migration of HSFBs 

**Figure 2:**
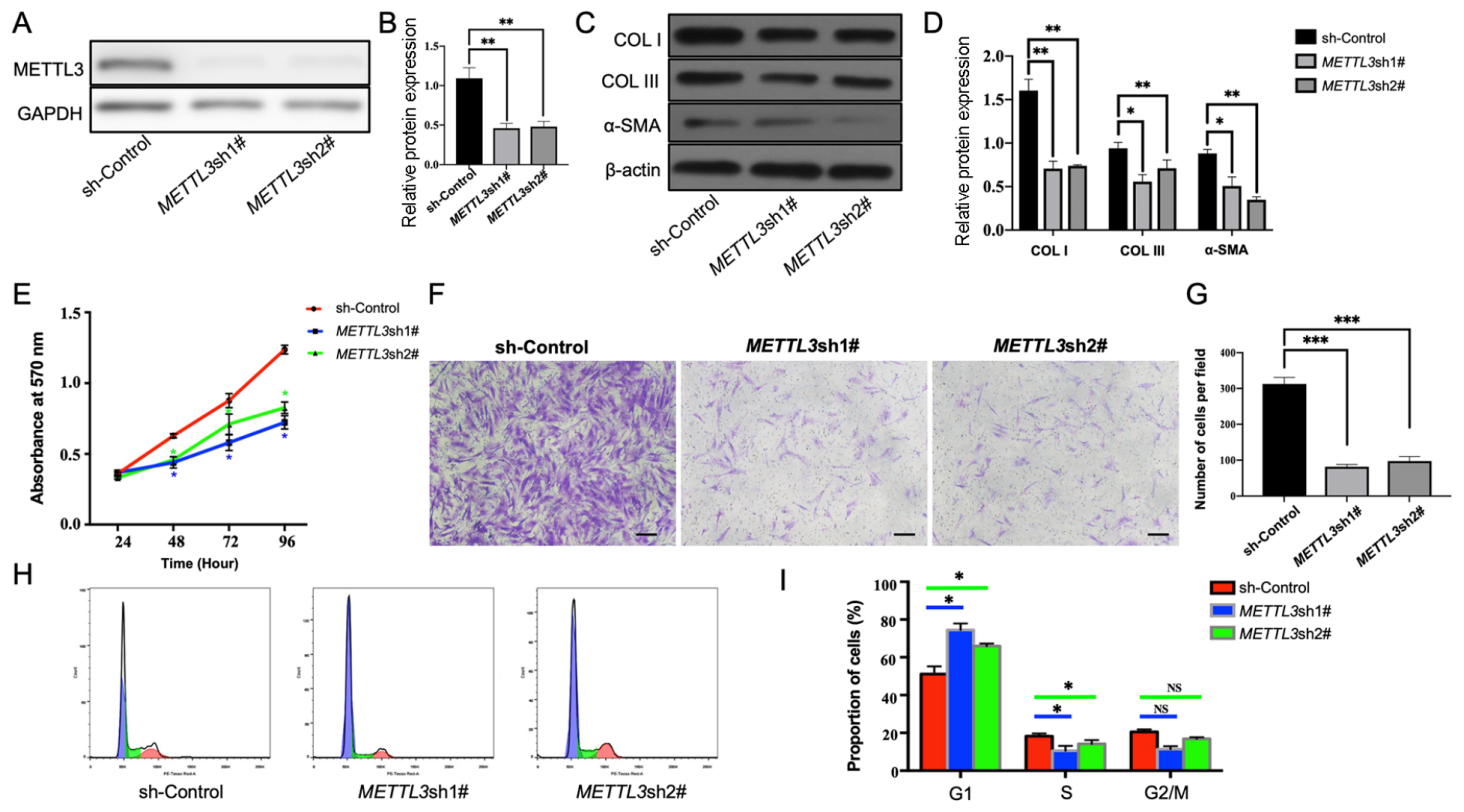
Figure 2 *METTL3* knockdown inhibits fibrosis, proliferation and migration in HSFBs (A) Western blot analysis showing reduced METTL3 levels in the METTL3 knockdown groups. (B) Grayscale analysis of METTL3 protein levels. (C) Western blot analysis showing decreased COL I/III and α-SMA levels in the METTL33 knockdown groups. (D) Grayscale analysis of COL I/III and α-SMA expression. (E) MTT assay was used to measure cell proliferation from 24 to 96 h in the METTL3-knockdown and control groups. (F) Transwell assays were used to assess cell migration in the METTL3-knockdown and control groups. (G) Quantification of migrating cells per field. (H,I) Flow cytometry analysis of the cell cycle distribution in the METTL3-knockdown group versus the control group. METTL3-knockdown groups: METTL3 sh#1 and METTL3 sh#2; control group: sh-Control. *P < 0.05, **P < 0.01, ***P < 0.001. Scale bar: 50 μm. HSFBs: hypertrophic scar-derived fibroblasts.

To investigate the effect of METTL3 on HSFBs, we knocked down *METTL3* using shRNA. As shown in Figure 2A,B, western blot analysis results showed significantly lower protein expression of METTL3 in *METTL3*-knockdown groups (METTL3sh1# and METTL3sh2#) than in the sh-Control group, indicating effective *METTL3* knockdown in HSFBs. *METTL3* knockdown also led to decreased protein expression of COL I/III and α-SMA ( Figure 2C,D). MTT assays indicated that *METTL3* silencing inhibited the cell proliferation of HSFBs ( Figure 2E). Transwell assays revealed a marked reduction in migration following *METTL3* knockdown ( Figure 2F,G). Additionally, *METTL3* knockdown in HSFBs resulted in G1 cell cycle arrest, as determined via flow cytometry analysis ( Figure 2H,I).

### METTL3 mediates m ^6^A-modification of pri-miR-31-5p in HSFBs 

**Figure 3:**
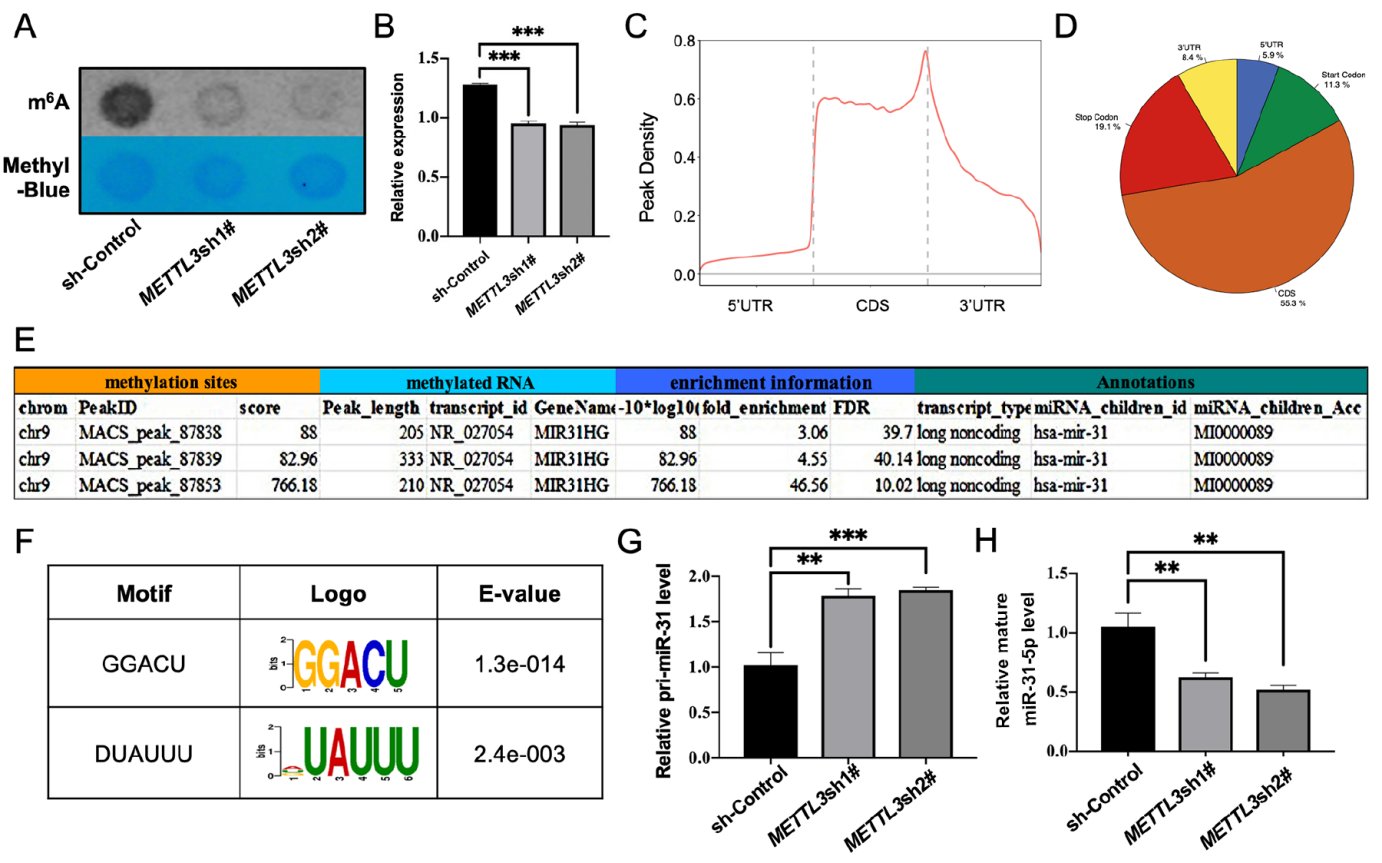
Figure 3 METTL3 mediates m ^6^A modification of pri-miR-31-5p in HSFBs (A) m6A dot-blot analysis for detecting m6A modification levels in HSFBs. (B) Grayscale analysis of the m6A dot-blot results. (C) Distribution of m 6A peaks across transcript regions (5′UTR, CDS, 3′UTR). (D) Distribution of m6A peaks. (E) m6A peak sites on pri-miR-31 in HSFBs. (F) Motif analysis of m6A peaks. (G) qRT-PCR results showing increased pri-miR-31 levels upon METTL3 knockdown. (H) qRT-PCR results showing decreased mature miR-31-5p levels upon METTL3 knockdown in HSFBs. CDS, coding sequence. **P < 0.01, *** P < 0.001.

m ^6^A dot blot analysis showed that *METTL3* knockdown significantly reduced m ^6^A levels in HSFBs ( Figure 3A,B). MeRIP-seq identified m ^6^A peaks predominantly in the coding sequence (CDS) region (55.3%) and the stop codon region (19.1%, Figure 3C,D). We identified m ^6^A modification sites on pri-miR-31 ( Figure 3E).

There was a high level of enrichment in the m ^6^A peaks of HSFB samples for the motifs GGACU and DUAUUU in HSFBs ( Figure 3F). *METTL3* knockdown resulted in upregulation of pri-miR-31 and downregulation of mature miR-31-5p ( Figure 3G,H), suggesting that METTL3 plays a role in regulating pri-miR-31 maturation via m ^6^A modification. 

### m ^6^A methylation regulates fibrosis and cell proliferation in fibroblasts via miR-31-5p 

**Figure 4:**
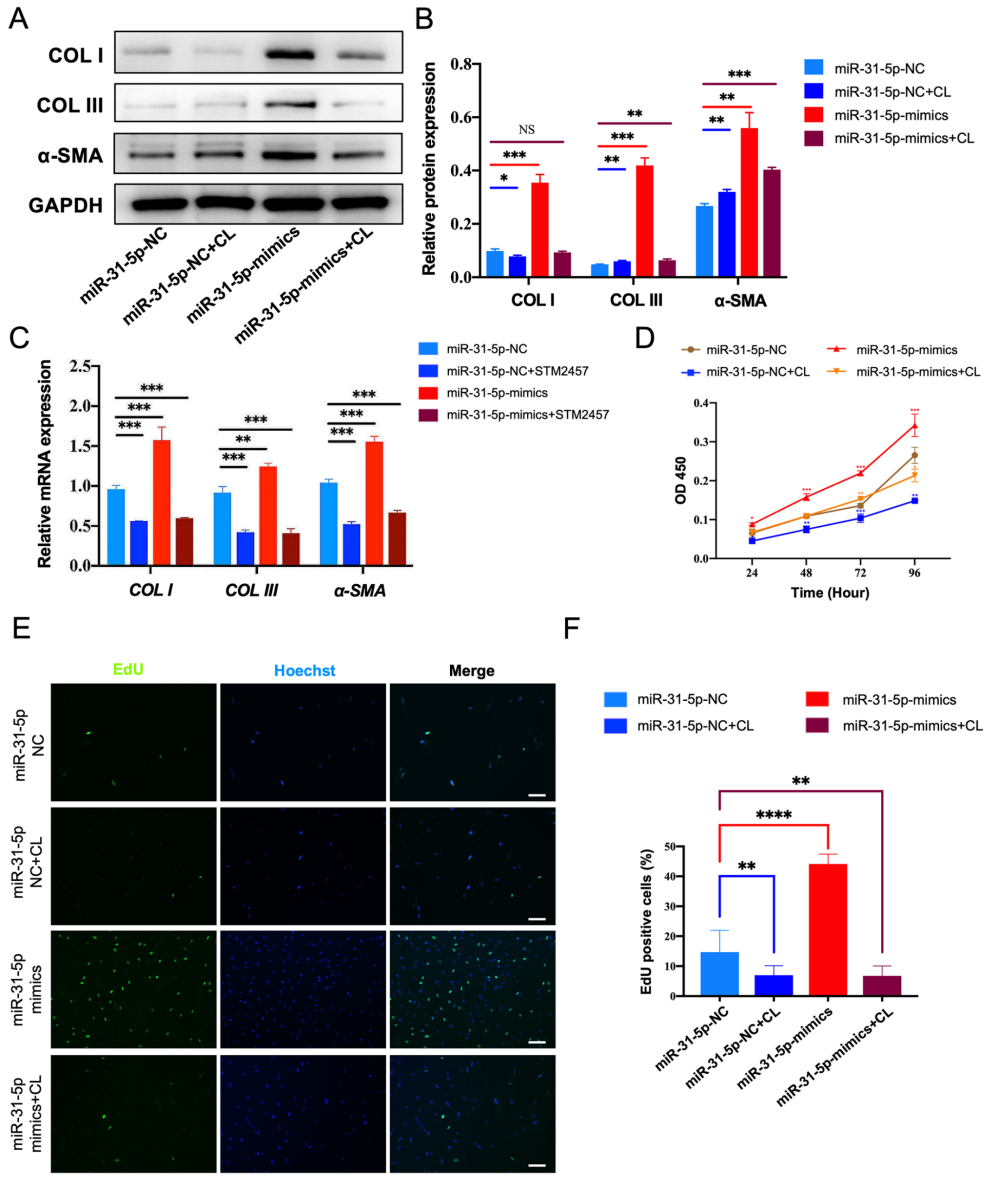
Figure 4 miR-31-5p counteracts the effects of m ^6^A methylation inhibitors on fibrosis and cell proliferation (A) miR-31-5p mimics partially reversed the m6A methylation inhibitor-mediated regulation of COL I/III and α-SMA in HSFBs. (B) Grayscale analysis of COL I/III and α-SMA levels. (C) miR-31-5p mimics partially attenuated the m6A methylation inhibitor-mediated regulation of cell proliferation in HSFBs. (D) Percentage of EdU-positive cells. (E) CCK-8 assay was used to measure the proliferative capacity of the cells from 24 to 96 h. (F) Percentage of EdU-positive cells. Groups: miR-31-5p-negative control (NC); miR-31-5p-NC + cycloleucine (CL, 40 μM) treatment; miR-31-5p-mimics; miR-31-5p-mimics + CL treatment. NS, not significant. **P < 0.01, ***P < 0.001, ****P < 0.0001. Scale bar: 50 μm.

To further elucidate whether miR-31-5p contributes to m ^6^A modification-mediated phenotypes in hypertrophic scars, we conducted reconstitution experiments with miR-31-5p mimics, the m ^6^A methylation inhibitor (CL) alone or with miR-31-5p mimics and CL in combination. Compared with the miR-31-5p NC group, the miR-31-5p NC+ CL group presented reduced expression of COL I/III and α-SMA in HSFBs. However, this effect was reversed by the miR-31-5p mimic ( Figure 4A,B). Similar results were observed in the fibroblasts treated with STM2457 (an METTL3 specific inhibitor) with or without miR-31-5p ( Figure 4C).

CCK8 assays showed that CL reduced the proliferation of HSFBs, and this effect was attenuated by the miR-31-5p mimic ( Figure 4D). The EdU assay also showed that the percentage of EdU-positive cells was significantly lower in the CL-treated group than in the control group, and this effect was attenuated by the miR-31-5p mimic ( Figure 4E,F **),** highlighting the critical role of miR-31-5p in the m ^6^A-mediated proliferation effects in HFSBs. 

### miR-31-5p inhibits ZBTB20 expression through targeting its 3′UTR

**Figure 5:**
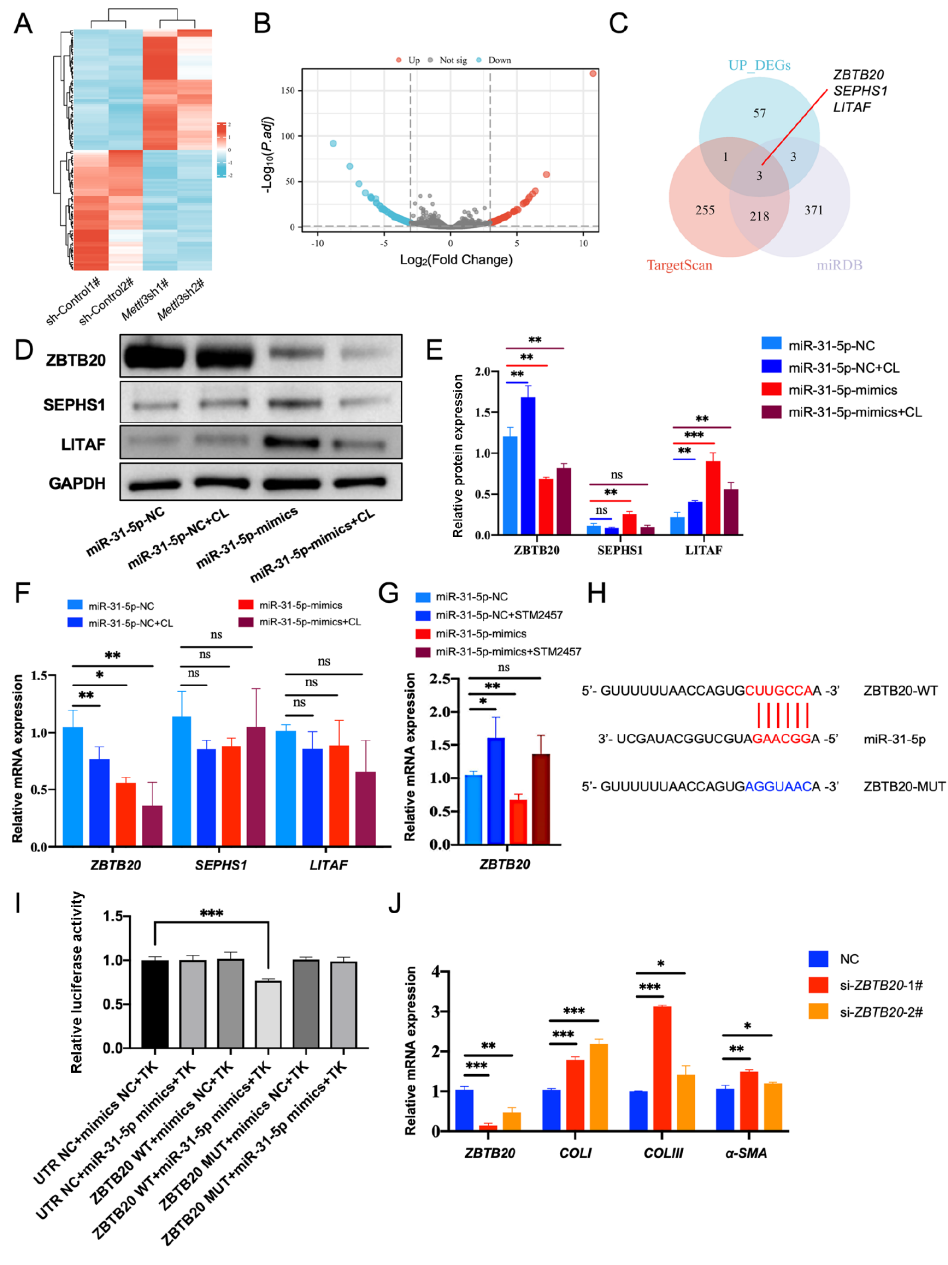
Figure 5 *ZBTB20* is a downstream target gene of miR-31-5p (A) Heatmap of genes differentially expressed between the METTL3-knockdown groups and the control groups. (B) Volcano map of genes differentially expressed between the METTL3-knockdown groups and the control groups. (C) Venn diagram illustrating the overlap between the miRDB and TargetScan predictions and upregulated genes in METTL3-knockdown cells. ZBTB20, LITAF and SEPHS1 are downstream target genes of miR-31-5p. (D) Western blot analysis of ZBTB20, LITAF and SEPHS1 protein levels. (E) Grayscale analysis of ZBTB20, LITAF and SEPHS1. (F) The mRNA levels of ZBTB20, LITAF and SEPHS1 were detected by qRT-PCR. Groups: miR-31-5p-negative control (NC); miR-31-5p-NC + cycloleucine (CL, 40 μM) treatment; miR-31-5p-mimics; miR-31-5p-mimics + CL treatment. (G) The METTL3 inhibitor STM2457 can suppress the mRNA levels of ZBTB20 mediated by the miR-31-5p mimic. Groups: miR-31-5p-negative control (NC); miR-31-5p-NC + STM2457 treatment; miR-31-5p-mimics; miR-31-5p-mimics + STM2457 treatment. (H) Information on the mutated sequence of ZBTB20 used for Dual-Luciferase reporter gene assays. (I) Dual-luciferase reporter gene assays confirmed that ZBTB20 is a downstream target gene of miR-31-5p. (J) Knockdown of ZBTB20 increases the mRNA levels of COL I/III and α-SMA. ZBTB20-knockdown groups: si-ZBTB20-1# and si-ZBTB20-2#; control group: negative control (NC). NS, not significant, *P < 0.05, **P < 0.01, ***P < 0.001.

To investigate the candidate targets underlying the effects of METTL3/m ^6^A/miR-31-5p on HSFBs, we performed RNA-seq on HSFBs from the *METTL3*-knockdown group and the control group. We analyzed the potential target genes by overlapping the RNA-seq results with the potential targets of miR-31-5p. Using |log2FC| > 3 and *P*_ adj_ value < 0.05 as thresholds, we obtained 75 downregulated genes and 64 upregulated genes ( Figure 5A,B). To predict the targets of miR-31-5p, we used the miRDB and TargetScan websites, which predicted 595 and 477 target genes, respectively. The predicted results were subsequently overlapped with upregulated genes in the *METTL3* knockdown group, and *ZBTB20* , *SEPHS1* and *LITAF* were identified as potential targets of miR-31-5p ( Figure 5C). qRT-PCR and western blot analysis confirmed that ZBTB20 expression was lower in the miR-31-5p mimic group than in the miR-31-5p-NC group ( Figure 5D‒F). STM2457 treatment also increased *ZBTB20* mRNA expression, and this effect was reversed by the miR-31-5p mimic ( Figure 5G). A dual-luciferase reporter gene assay indicated that miR-31-5p mimics significantly reduced luciferase activity in the ZBTB20-WT group but had no effect on the ZBTB20-MUT group ( Figure 5H,I). Moreover, *ZBTB20* knockdown decreased the expression of COL I/III and α-SMA in fibroblasts ( Figure 5 J).

## Discussion

HS is an abnormal scar characterized by fibroblast hyperproliferation and excessive extracellular matrix deposition. Drugs such as steroids and 5-fluorouracil have been used to treat HS [16], and recently, vitamin D3 and related derivatives showed the ant-fibrotic activities on human and murine fibroblasts [17]. However, the specific treatments for HS are still not well established [18], as the underlying regulatory mechanisms are complex. We reported that miR-31-5p is highly expressed and significantly correlated with excessive deposition of ECM and fibrosis in HS [13]. This study extends these findings by demonstrating that the m ^6^A methyltransferase METTL3 is overexpressed in HS tissues and promotes the maturation of pri-miR-31, suggesting a potential regulatory mechanism in HS formation. 

Compared with that in NS tissues, the m ^6^A modification level in HS tissues is significantly increased, indicating that post-transcriptional modification of RNA plays a crucial role in HS formation. This finding aligns with the findings of Liu *et al* . [9], who reported 14,791 new m ^6^A peaks and 7,835 lost peaks in HS tissues, highlighting an overall increase in the m ^6^A level. While Liu’s study systematically analyzed the m ^6^A modification pattern, the specific molecular mechanisms involved remain unclear. Our data confirmed that METTL3 was elevated in HS and that *METTL3* knockdown reduced the expressions of the profibrotic proteins COL I/III and α-SMA, inhibited proliferation and migration ability and induced G1 cell cycle arrest, suggesting that METTL3 plays a role of in HS formation. 

Silencing of *METTL3* alters miR-31-5p expression in HSFBs by reducing the m ^6^A modification of pri-miR-31. METTL3 is known to regulate disease progression by promoting pri-microRNA processing and maturation in an m ^6^A-dependent manner [ 19 ‒ 21]. Overexpression of miR-31-5p in NSFBs increases the expressions of profibrotic proteins (COL I/III and α-SMA) and proliferation, whereas the m ^6^A inhibitor CL reduces these effects. 

These findings suggest that m ^6^A methylation regulates miR-31-5p function by inhibiting pri-miR-31 maturation in fibroblasts. After *METTL3* knockdown, mature miR-31-5p levels decreased in HSFBs, leading to upregulation of target gene expression. RNA-seq and miRNA target gene prediction tools identified *ZBTB20* , *SEPHS1*, and *LITAF* as potential downstream targets. Validation through qRT-PCR and western blot analysis confirmed that only ZBTB20 was significantly reduced in the miR-31-5p mimic-treated NSFBs. The dual-luciferase reporter assay results indicated that miR-31-5p directly targets *ZBTB20*. This study identified *ZBTB20* as a direct functional target of miR-31-5p in HSFBs. 

ZBTB20 (zinc-finger and BTB domain-containing 20), a member of a zinc-finger protein subfamily, includes C2H2 Krüppel-type zinc-fingers and a BTB/POZ domain [22]. ZBTB20 is crucial for the differentiation and function of various cell types, including those involved in the liver, immune system and glucose/lipid metabolism. [22]. It has been reported that ZBTB20 is downregulated in myofibroblasts during renal fibrosis [23], suggesting a negative correlation with fibrosis progression. However, the function of ZBTB20 in skin fibroblasts remains unclear. Our data show that *ZBTB20* knockdown reduces the expressions of COL I/III and α-SMA in fibroblasts, indicating that the m ^6^A-dependent METTL3/miR-31/ZBTB20 axis facilitates hypertrophic scar fibrosis. 

Nevertheless, there are several limitations in our study. First, the present study did not investigate how METTL3 overexpression affects hypertrophic scarring. Second, the precise molecular mechanisms by which ZBTB20 regulates HS fibrosis warrant further investigation. In a summary, the METTL3/m ^6^A/miR-31-5p/ZBTB20 axis represents a potential regulatory mechanism in HS development. Targeting this pathway may provide new therapeutic strategies for the treatment of HS. 
